# Irf5 Knockdown in Bone Marrow-Derived Macrophages Favors M1-to-M2 Transition

**DOI:** 10.3390/cells15030238

**Published:** 2026-01-26

**Authors:** Elizaveta Petrova, Ekaterina Sherstyukova, Snezhanna Kandrashina, Vladimir Inozemtsev, Alexandra Tsitrina, Viktoriya Fedorova, Mikhail Shvedov, Artem Kuzovlev, Maxim Dokukin, Yuri Kotelevtsev, Arsen Mikaelyan, Viktoria Sergunova

**Affiliations:** 1 Odintsovo Center of Medical and Biological Technologies, 143025 Moscow, Russia; 01lpetrova12@gmail.com (E.P.); v.fedorova@skoltech.ru (V.F.); 2 Federal Research and Clinical Center of Intensive Care Medicine and Rehabilitology, V.A. Negovsky Research Institute of General Reanimatology, 107031 Moscow, Russia; kmanchenko@yandex.ru (E.S.); snezhanna.lyapunova@yandex.ru (S.K.); va.inozemcev@physics.msu.ru (V.I.); shvedovmike@bk.ru (M.S.); artem_kuzovlev@fnkcrr.ru (A.K.); safmlab@gmail.com (M.D.); 3 Ilse Katz Institute for Nanoscale Science and Technology, Ben-Gurion University of the Negev, Beer Sheva 8410501, Israel; 4 Koltzov Institute of Developmental Biology of the Russian Academy of Sciences, 26 Vavilov Street, 119334 Moscow, Russia; a.mikaelyan@idbras.ru

**Keywords:** macrophages, polarization, M1, M2, IRF5, siRNA, mitochondria, biomechanics, Young’s modulus, atomic force microscopy

## Abstract

The transcription factor IRF5 maintains macrophages in the pro-inflammatory M1 state. We assessed the effects of siRNA-mediated knockdown of Irf5 on murine bone marrow-derived macrophages (BMDM) in M0, M1 and M2 states. Knockdown of Irf5 in M1 macrophages made them phenotypically similar to M2 macrophages, which was reflected in the decreased expression of the M1 marker iNOS, increased expression of the M2 marker CD206, increased mitochondrial content and respective morphological changes. Interestingly, the M2 phenotype was also affected by the reduction in Irf5. Using atomic force microscopy (AFM), we showed that Irf5 knockdown increases plasma membrane roughness, particularly in M2 macrophages. AFM-based stiffness measurements indicated that Irf5 knockdown altered macrophage elasticity, potentially influencing their functional behavior. Our data suggest a complex role of IRF5 in macrophage polarization, supporting its dual role as a transcriptional activator and repressor both in M1 and M2 states, and highlight the importance of IRF5 in the maintenance of metabolic and functional properties of macrophages.

## 1. Introduction

Macrophages are immune cells that regulate both innate and adaptive immune responses and play a critical role in inflammation. Being highly versatile, macrophages can adopt different functional states, commonly categorized as classically (M1) and alternatively (M2) activated phenotypes [1,2]. M1 macrophages produce pro-inflammatory cytokines, such as TNF-α, IL-1, IL-6, IL-12 and IL-23, to initiate and sustain inflammation. They are effective at eliminating pathogens through phagocytosis and the production of reactive oxygen species (ROS) and nitric oxide (NO) and facilitate antigen presentation to activate the adaptive immune response. In contrast, M2 macrophages have anti-inflammatory functions. By secreting anti-inflammatory cytokines like IL-10 and TGF-β, M2 macrophages resolve inflammation and promote tissue healing and remodeling. Moreover, M2 macrophages modulate the immune response, ensuring it remains balanced and non-destructive [3]. M1 macrophages can change their functional state into M2 and vice versa [4].

The family of interferon regulatory factors (IRFs) plays an important role in regulating macrophage polarization to M1 or M2 phenotypes [5]. Interferon regulatory factor 5 (IRF5) is a transcription factor that activates the expression of type I interferons (IFN) IFNα and IFNβ, as well as inflammatory cytokines downstream of endo-lysosomal toll-like receptors TLR7, TLR8 and TLR9 [6]. IRF5 enhances the expression of pro-inflammatory cytokines (IL-12 and IL-23) and suppresses the expression of anti-inflammatory cytokine IL-10 in human M1 macrophages [7]. Activation of IRF5 occurs through phosphorylation by IκB kinase β in response to TLR signaling, after which IRF5 translocates to the nucleus and initiates the transcription of genes involved in the innate immune response [6,8]. IRF5 is implicated in a wide range of acute and chronic inflammatory conditions [9,10,11,12,13]. Importantly, IRF5 was shown to be directly associated with the macrophage activation syndrome (MAS), which is observed in conditions like systemic juvenile idiopathic arthritis [10]. MAS is a life-threatening condition that is characterized by extremely high levels of pro-inflammatory cytokines.

Earlier studies showed that the knockout of IRF5 is associated with an increase in M2 macrophages and elevated levels of anti-inflammatory cytokines in animal models of inflammatory conditions, such as liver fibrosis, asthma, and systemic lupus erythematosus (SLE), where M1 macrophages tend to be predominant [14,15,16]. This led to the suggestion that IRF5 is an attractive therapeutic target, which regulates the switch from M1 to M2 phenotype [17]. Several small molecule inhibitors of IRF5 have already been identified. YE6144 is an agent that prevents the phosphorylation of IRF5; it was already shown to be promising in the treatment of SLE [18]. Meanwhile, feeblin interferes with the interaction between the adaptor protein TASL and the endolysosomal transporter SLC15A4, which are upstream to IRF5 in the TLR signaling pathway, to prevent the activation of the IRF5 protein [19]. At the moment, the potential of these and other similar agents is being actively explored.

To model the effects of small molecule inhibitors of IRF5, we performed a knockdown of the *Irf5* gene in mouse bone marrow-derived macrophages (BMDM) using siRNA against Irf5 (siIrf5) which was developed in our laboratory and described earlier [20]. Using fluorescence microscopy, we assessed changes in the expression of M1 and M2 markers and quantified mitochondrial content. Atomic force microscopy was used to evaluate the mechanical properties of cells and to visualize the nanostructure of the plasma membrane. A combination of these two methods was applied for the assessment of cell morphology. Our results show that the knockdown of Irf5 changes the phenotypic characteristics in both M1 and M2 polarization states. We demonstrate for the first time that Irf5 knockdown together with the alteration in the expression of M1 and M2 markers is associated with changes in the biomechanical properties of the cells.

## 2. Materials and Methods

### 2.1. Preparation of L929 Cell-Conditioned Medium (LCCM)

The L929 cells were kindly provided by I.M Bogdanova (Research Institute of Human Morphology, Moscow, Russia) and were maintained in DMEM (Thermo Fisher Scientific, Waltham, MA, USA) supplemented with 10% FBS (Thermo Fisher Scientific, Waltham, MA, USA), 1% GlutaMax (Thermo Fisher Scientific, Waltham, MA, USA) and 1% penicillin–streptomycin (Gibco, Thermo Fisher Scientific, Waltham, MA, USA), at 37 °C and 5% CO_2_. The medium from L929 cells was purified by centrifugation at 3700 RPM using Centrifuge 5804 R (Eppendorf, Hamburg, Germany), after which it was stored at −80 °C in aliquots.

### 2.2. Mouse Bone Marrow Cells Isolation and Differentiation into Bone Marrow-Derived Macrophages (BMDM)

The animal study protocols were approved by the Ethics Committee of the Koltzov Institute of Developmental Biology of the Russian Academy of Sciences (protocol number: 55; 9 December 2021). The primary culture of BMDM was produced from the bone marrow of 12–16-week-old FVB mice as described [21]. The animals were euthanized by cervical dislocation and were soaked in 70% ethanol. After that, the animals’ hind legs were cleared from fur and skin and detached from the hip joint with scissors, ensuring that the femur and the tibia of each limb remained intact. The detached limbs were also soaked in 70% ethanol to prevent contamination from blood and intestines. The tibia and femurs were cleared from muscle, tendons and fat using forceps and scissors.

After rinsing the bones in sterile PBS containing 1% penicillin–streptomycin (Gibco, Thermo Fisher Scientific, Waltham, MA, USA), the bone marrow was extracted by removing the bones’ epiphyses from both sides with scissors and injecting sterile PBS with an insulin syringe (26G needle, SFM Hospital Products GmbH, Berlin, Germany) into the bone to flush and collect the contents of the bone. After the bone marrow was collected, it was centrifuged at 1100 RPM using the LMC-3000 centrifuge (Biosan, Riga, Latvia) for five minutes. The supernatant was discarded and the pellet containing the bone marrow was resuspended in the growth medium: DMEM-F12 (Thermo Fischer Scientific, Waltham, MA, USA) supplemented with 10% FBS (Thermo Fisher Scientific, Waltham, MA, USA), 1% GlutaMax, 1% penicillin–streptomycin (Thermo Fisher Scientific, Waltham, MA, USA), LCCM (30% *v*/*v*). The cells were seeded into flasks with the surface area of 175 cm^2^ and were cultured for 7 days at 37 °C and 5% CO_2_.

### 2.3. Polarization and Transfection of BMDM

Prior to polarization and transfection, BMDM were detached from the culture flask surface using a scraping tool and were seeded into 96-well plates, with the seeding density being 7 × 10^4^ cells per well, to achieve 80% confluency. To polarize the BMDM toward the M1 and M2 phenotype, the cells were incubated with respective polarizing agents for 24 h: M1: 100 ng/mL *E. coli* LPS (Sigma-Aldrich, St. Louis, MO, USA); M2: 20 ng/mL IL-4 and 20 ng/mL IL-10 (Miltenyi Biotec, Bergisch Gladbach, Germany). The M0 cells did not receive any additional treatment. For transfection experiments, the siRNA particles (both siIrf5 and siLuc) were combined with Lipofectamine RNAi Max (Invitrogen, Carlsbad, CA, USA), which served as a carrier, as described [20]. After that, the siRNA particles were added to the respective groups, with the final concentration being 10 nM. The overall duration of transfection was 24 h. The siRNA against Irf5 had the following sequence: AAGAAuGGccuGAuGucAATsT (sense), UUGAcAUcAGGCcAUUCUUTsT (antisense).

### 2.4. Western Blot Analisis

BMDM cells was lysed in RIPO lysis buffer (Thermo Fisher Scientific, USA) containing protease inhibitor cocktail (cOmpelet, Roche, Mannheim, Germany). Cell lysates (50 µg protein per lane) were resolved on 8% SDS-PAGE and transferred onto nitrocellulose membrane (Bio-Rad, Feldkirchen, Germany). The membranes were blocked 5% not-fat milk and incubated with the following primary antibodies: anti-IRF5 (1:500, E-AB-19512, Elabscience, Houston, TX, USA) and anti-β-Actin (1:5000, A5541, Sigma-Aldrich, USA). HRP-conjugated anti-mouse (1:5000, 31430, Invitrogen, USA) or anti-rabbit IgG (1:5000, 31466, Invitrogen, USA) were used as secondary antibodies. The reactive bands were detected using SuperSignal West Pico PLUS Chemiluminescent Substrate (34577, Thermo Fisher Scientific, USA) on the Vilber Lourmat Fusion Solo S imaging system (Vilber, Collégien, France). The bands intensity was quantified by using ImageJ 1.54g software. GraphPad Prism 8 software was used for statistical analyze and graphs.

### 2.5. Staining and Imaging Procedures

After the cells underwent polarization and transfection, one group of cells was fixed for immunocytochemistry experiments and stored at 4 °C, while the other group was maintained live for visualization of mitochondria or atomic force microscopy experiments. All cells were visualized with the use of the Leica DMi8 THUNDER imager (Leica, Wetzlar, Germany), with the following settings: image format: Bin 2 × 2, mode: low cell noise, image depth: 8-bit. For statistical analysis, images were acquired using 20× and 40× air lenses, with a minimum of 4 images per group. For morphological visualization, a 63× oil-immersion lens was used.

### 2.6. Visualization of Mitochondria

In the group, where the cells underwent live staining, the cells were treated with a growth medium containing the MitoFlamma Green dye (RMS1101, 1:1000, BioActs, Incheon, Republic of Korea) for 50 min at 37 °C and 5% CO_2_, as described [22]. After that, the medium was replaced with PBS. The images (n = 10) were acquired as described above with the overall duration of imaging being 1 h.

### 2.7. Immunocytochemistry

The cells were fixed with 10% buffered formalin (Biovitrum, Saint Petersburg, Russia) for 15 min (RT) and were washed with PBS three times (3 min per wash, RT). After that, the cells were pre-incubated in PBS containing 1% *w*/*v* BSA and 0.4% Triton X-100 for 30 min (RT). The samples were incubated in respective primary antibody solutions overnight at 4 °C. The following primary antibodies were used: iNOS (rabbit polyclonal, 1:100, Abcam, Cambridge, UK), CD206 (rabbit polyclonal, 1:200, Abcam) [23,24]. After that the samples were washed with PBS three times and incubated in the secondary antibody solution for 1 h (RT); the solution contained the Alexa Fluor-conjugated donkey anti-rabbit secondary antibody (λ = 647 nm, 1:1000, Invitrogen), FSD Fluor™ 555 Phalloidin (1:1000, BioActs, Incheon, Republic of Korea) and Hoechst dye (1:2000, Thermo Fischer Scientific, Waltham, MA, USA) [25]. Both the primary and secondary antibodies were diluted in PBS containing 1% *w*/*v* BSA and 0.4% Triton X-100. After that the samples were washed with PBS three times (3 min per wash, RT) and then mounted in Abberior mount solid antifade (Abberior, Göttingen, Germany) on glass slides prior to imaging. Samples that were incubated with secondary antibodies only served as the negative control. For statistical analysis, images were acquired using the 20×/NA 0.80 Plan Apochromat air lens. For morphological visualization, a 63×/NA 1.40 Plan Apochromat oil-immersion lens was used.

### 2.8. Image Quantification

The images of MitoFlamma Green-stained BMDM were analyzed using the QuPath software (version 0.5.0) [26], where composite images were used for the creation of a custom classifier, which allowed to distinguish the cells from the background. After that, a binary mask that was created based on the cell outlines from the composite image was imposed onto the fluorescent image of mitochondria to determine the mean fluorescence of individual cells. Fluorescent images of each marker were acquired, with each group imaged with the same exposure and laser intensity.

Meanwhile, the fluorescent images of M1 and M2 markers, and the phalloidin stain were analyzed using the Cell Profiler software (version 4.0.5) [27]. For the analysis of images of the iNOS stain, the respective images of the DAPI stain were smoothed by applying the median filter; the smoothed images were used for the identification of primary objects (i.e., nuclei). Prior to the identification of secondary objects (i.e., cells stained against iNOS), the brightness of the respective images were adjusted using image math and were smoothed using the Gaussian filter. The cells that were stained against iNOS were identified by applying the propagation algorithm to the image of nuclei. The data about the mean fluorescence intensity were acquired from these images [27].

The images of the CD206 stain were analyzed using a similar algorithm; however, there was no smoothing prior to the identification of cell nuclei. These images were used for the extraction of data about the mean fluorescence of the CD206 stain. Images of the phalloidin stain were quantified to acquire data about the morphology descriptors (i.e., major axis length, minor axis length and form factor (circularity)).

### 2.9. Atomic Force Microscopy

NTEGRA Prima and NTEGRA BIO atomic force microscopes (NT-MDT SI, Moscow, Russia) were used for cell surface imaging. Regular tapping NSG01 cantilevers (NT-MDT SI, Moscow, Russia) with a tip radius of ~10 nm and spring constant of 5 N/m were utilized. All images were collected with the resolution of 512 × 512 or 1024 × 1024 pixels and size from 5 × 5 μm^2^ to 100 × 100 μm^2^. The lateral scanning speed was 0.3 Hz. All surface maps were imaged in ambient conditions with the relative humidity of 35–50%. Prior imaging all cells were fixed with 4% paraformaldehyde for 30 min at room temperature and washed three times in 1 × PBS. To remove salts, cells were rinsed in distilled water for 1 min; this procedure was repeated twice. All samples were dried in air for 2 h before transferred to AFM.

The mechanical properties of the cells were evaluated from the force curves obtained by AFM. The elastic modulus (Young’s modulus) value was used to compare different cell types. The elastic modulus is hardware-independent and allows comparing cells measured on different microscopes. In order to assess the correctness of the obtained data, 2 types of probes were used in this study: SD-R150-T3L450B (Nanosensors, Switzerland) with parameters: spring constant 1 N/m and tip radius 150 nm; and SD-Sphere-CONT–L–10 (Nanosensors, Switzerland) with parameters: spring constant 0.2 N/m and tip radius 2000 nm. All measurements were performed on the living cells without prior preparation, with the overall imaging duration being 6 h. Measurements were performed at room temperature 24 °C in 1× PBS solution.

Before each experiment, the cantilever was fully calibrated. Vertical deflection sensitivity was calibrated against clean glass slide in 1× PBS. Cantilever spring constant was checked before experiment using the Sader method. All force–distance curves were collected at fixed vertical scanner speed of 5 μm/s.

AFM images of the cell surface maps were processed using Image Analysis software version 3.5 (NT-MDT SI, Russia) and Gwyddion version 2.65 [28].

### 2.10. Statistical Analysis: Fluorescent Microscopy

All data are represented as mean values ± standard error (SEM). Statistical evaluation of differences between the mean fluorescence intensity of markers of M1 and M2 polarization or the MitoFlamma Green dye, circularity, elongation factor, and the Young’s modulus of each group was done by performing pairwise comparisons (Mann–Whitney U test). The data was acquired in the form of csv files and analyzed using the scipy.stats module from the Scipy library (Python version 3.13.2). The changes with *p* < 0.05 were considered statistically significant.

### 2.11. Statistical Analysis: AFM

Images of 10 typical cells were obtained for each sample type. High-resolution images of cells were obtained to analyze changes in the local structure of the macrophage surface. For this purpose, 5 × 5 maps with a resolution of 512 × 512 points were measured. To analyze the biomechanical properties, 50 cells were measured for each sample. For the analysis of mechanical properties, data from about 450 cells were analyzed.

Statistical analysis of the obtained results was performed using the OriginPro 2019 (v.9.6.0.172) program (OriginLab Corporation, Northampton, MA, USA). Statistics are presented as mean with standard deviation (mean ± SD). The non-parametric Mann–Whitney test was used to check the significance of the difference between the data and the control value. Differences were considered statistically significant at * *p* < 0.05; ** *p* < 0.01; *** *p* < 0.001.

## 3. Results

### 3.1. IRF5 Has Reverse Effects on Expression of iNOS and CD206 in M1 and M2 Macrophages

**Figure 1:**
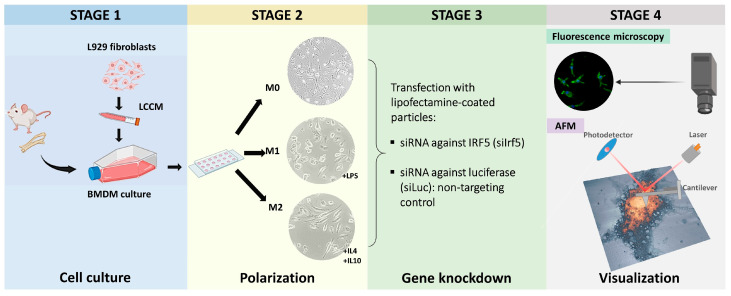
An overview of the experimental procedures. Stage 1 (Cell culture): Isolation of mesenchymal stem cells and acquisition of primary macrophage culture (BMDM), with purified L929 cell-conditioned medium (LCCM) being the source of macrophage colony-stimulating factor (M-CSF). Stage 2 (Polarization): Polarization of macrophages to M1 (100 ng/mL *E. coli* LPS) and M2 (20 ng/mL IL-4 and 20 ng/mL IL-10) states for 24 h. Stage 3 (Gene knockdown): Transfection (24 h): siRNA complementary to IRF5 (siIrf5), siRNA complementary to luciferase (siLuc)-non-targeting control. Stage 4 (Visualization): Data acquisition: fluorescence microscopy and atomic force microscopy.

Figure 1 shows a diagram of the experimental procedures. We first identified the most effective siRNA for Irf5 silencing using the RAW 264.7 murine macrophage cells line (Figure S5). Selected siRNA has been evaluated for effects of Irf5 knockdown on macrophage polarization.

**Figure 2:**
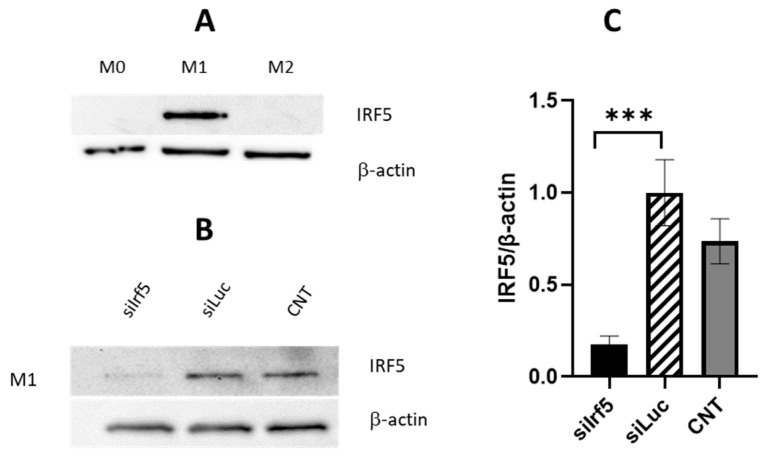
Western blot analysis of IRF5 expression in bone marrow-derived macrophages (BMDM): (**A**) IRF5 expression in BMDM maintained in cell culture (M0) and polarized for 24 h to M1 phenotype with LPS, or to M2 phenotype with Il6 and Il10. (**B**) Expression of IRF5 in M1 macrophages 48 h post transfection with IRF5 specific siRNA. (**C**) Bar graph displayed IRF5 protein quantity normalized to β-actin compared to cells untreated and treated with luciferase specific siRNA. CNT—untreated cells (mean ± SD, n = 3, *** *p* ≤ 0.001).

Next, we estimated protein expression IRF5 in BMDMs under different polarization conditions. Irf5 was specifically induced for M1 polarization, but not M2 (Figure 2A). Treatment with Irf5-specific siRNA significantly reduced IRF5 protein level, whereas non-targeted control luciferase siRNA had no effect (Figure 2B,C).

**Figure 3:**
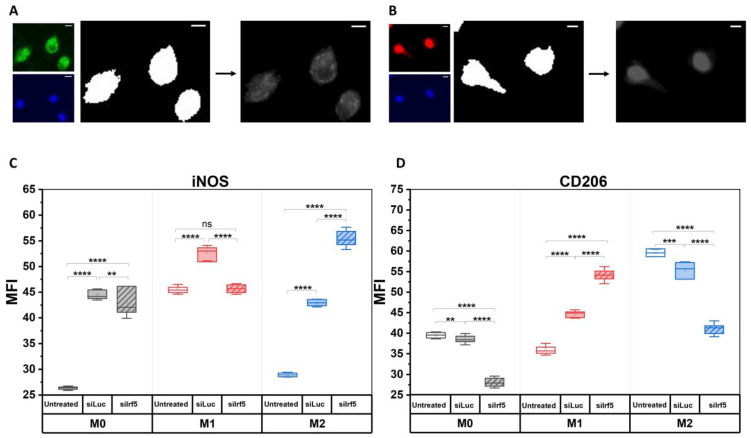
Changes in expression of iNOS and CD206 in response to IRF5 knockdown. Fixed BMDM were stained against iNOS and CD206, in combination with Hoechst stain to visualize DNA. Fluorescent images of untreated, siLuc-treated and siIrf5-treated M0, M1 and M2 macrophages were acquired and quantified to assess levels of mean fluorescence intensity. Image analysis of iNOS stain (green, (**A**)) and CD206 stain (red, (**B**)) combined with Hoechst stain (blue): fluorescent images were thresholded to create binary masks, which were imposed on the respective fluorescent images to extract data about mean fluorescence intensity (MFI), magnification: ×20, scale bar = 20 µm ((**C**,**D**), respectively) (M0—grey, M1—red, M2—blue). Mann–Whitney U test, ns—not significant, ** *p* < 0.01, *** *p* < 0.001, **** *p* < 0.0001.

We also performed immunofluorescence staining of M0, M1 and M2 macrophages that were treated with siIrf5, using iNOS and CD206 as markers of M1 and M2 polarization, respectively (Figure S1) [29,30]. Fluorescent images of iNOS and CD206 stains were thresholded to generate masks, which were applied to the respective fluorescent images to extract data about the mean fluorescence intensity (MFI) of stains within each experimental group (Figure 3A,B). In this and all subsequent experiments, siRNA against luciferase (siLuc) was used as a non-targeting control.

The MFI of iNOS in M0 and M2 macrophages increased significantly after treatment with siLuc, while the MFI of CD206 decreased. In M1 macrophages, we also observed an increase in the MFI of iNOS in response to treatment with siLuc, although it was less sharp, as it was initially higher in the untreated M1 macrophages as compared to untreated M0 and M2 macrophages. Moreover, we observed an increase in the MFI of CD206. However, treatment with siIrf5 led to a slight decrease in the MFI of iNOS in M0 macrophages and a strong decrease in M1 macrophages, while in M2 macrophages, the MFI of iNOS increased nearly 1.5-fold. At the same time, we observed a sharp decrease in the MFI of CD206 in M0 and M2 macrophages after treatment with siIrf5, while in M1 macrophages, the MFI of CD206 increased (Figure 3C,D). These results allow to conclude that IRF5 enhances the expression of a key pro-inflammatory effector iNOS in M1 state and suppresses its expression in M2 state. On the other hand, the pattern is reversed with the functional anti-inflammatory M2 marker, CD206; IRF5 suppresses its expression in M1 and enhances in M2. This indicates that IRF5 plays a complex role in regulating the macrophage expression patterns in different polarization states. Hence, inhibition of IRF5 may not only suppress the pro-inflammatory effectors in M1 macrophages, but simultaneously may compromise the anti-inflammatory M2 state.

### 3.2. Knockdown of Irf5 in M1 Macrophages Brings up the Mitochondria Content to M2 Level

**Figure 4:**
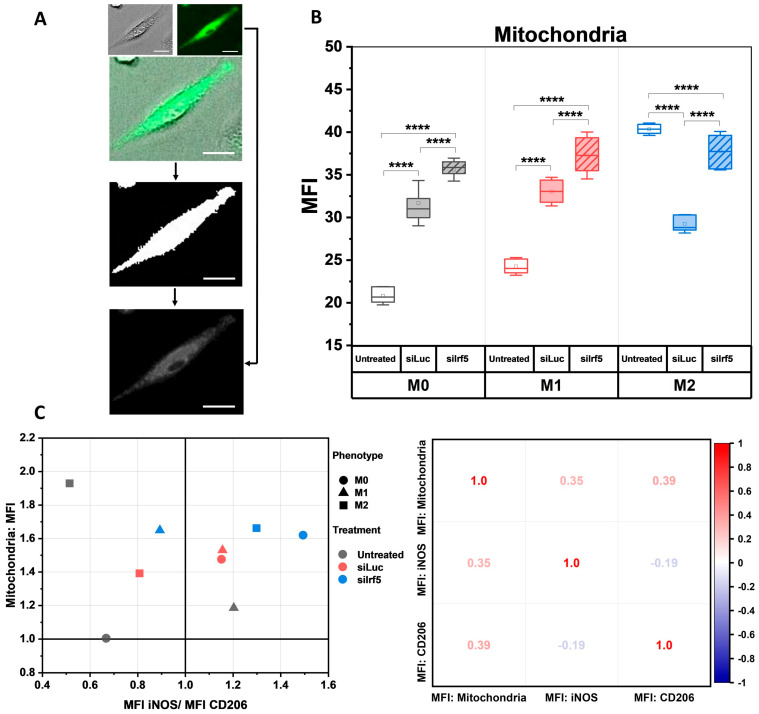
Changes in mitochondrial content after knockdown of Irf5 gene. Live cells were stained with fluorescent MitoFlamma Green dye. Brightfield (grey) and fluorescent images (green) of untreated, siLuc-treated and siIrf5-treated M0, M1 and M2 macrophages were acquired; merged (brightfield + fluorescent) images were used to create masks, which were then applied to the respective fluorescent images of mitochondria to extract intensity values corresponding to individual cells (**A**). Mean fluorescence intensity (MFI) of mitochondria stained with MitoFlamma Green stain. Magnification: ×20, scale bar = 20 µm (**B**). Association between functional state of macrophages and their mitochondrial content. Mean fluorescence intensity (MFI) values of iNOS, CD206 and mitochondria stains were normalized to the lowest value, which corresponded to the MFI of stained mitochondria in the untreated M0 group. Normalized values were used to construct the iNOS/CD206 MFI ratio (shown on the *X*-axis), where values greater than 1 indicated a pro-inflammatory M1 phenotype, whereas values below 1 corresponded to an anti-inflammatory M2 phenotype. Normalized MFI of MitoFlamma Green stain are shown on the *Y*-axis (**left**). Correlation between MFI of iNOS, CD206 and mitochondria (**right**) (**C**). Mann–Whitney U test, **** *p* < 0.0001.

Due to their distinct functional roles in immune response, there is a difference between the metabolic adaptations of M1 and M2 macrophages. M1 macrophages mainly rely on glycolysis, while M2 macrophages depend on oxidative phosphorylation. Therefore, M2 macrophages have a higher mitochondrial content as compared to M1 macrophages [31]. We decided to investigate whether the knockdown of the *Irf5* gene had any effect on the mitochondrial content. Live M0, M1 and M2 macrophages from all experimental groups were stained with the fluorescent MitoFlamma Green dye, which is used for the visualization of mitochondria (Figure S2). We also acquired brightfield images of the cells, which were merged with the fluorescent images to create masks, which corresponded to the outlines of the individual cells. These masks were applied to the respective fluorescent images of mitochondria to extract the intensity values corresponding to individual cells (Figure 4A). As the MitoFlamma Green dye is not dependent on the mitochondrial membrane potential, the brightness of each cell was directly proportional to its mitochondrial content.

In untreated cells, the mitochondrial content was the highest in M2, as compared to M1 and M0 macrophages. In M0 and M1 macrophages, the MFI of mitochondria increased after treatment with siLuc. Treatment with siIrf5 was followed by an even sharper increase in the MFI of M0 and M1 cells. Meanwhile, the MFI of M2 macrophages decreased in response to treatment with siLuc and increased in response to treatment with siIrf5 to the level of those that were not treated (Figure 4B). These findings demonstrate that the expression of IRF5 restricts the mitochondrial content in M1 macrophages to a lower level. The increase in mitochondrial content of M1 macrophages following depletion of IRF5 brings their metabolic phenotype closer to that of M2 macrophages, which further supports the role of IRF5 in promoting a pro-inflammatory M1 state while suppressing the anti-inflammatory M2 state (Figure 4C).

### 3.3. Knockdown of Irf5 in M1 Macrophages Makes Them Morphologically Similar to M2 Macrophages

Polarized macrophages have distinct morphological characteristics. M1 macrophages have a so-called “fried egg” or “pancake” appearance, while M2 macrophages have an elongated spindle-like shape, which has been demonstrated both in vitro and in vivo [32,33,34]. We hypothesized that the knockdown of Irf5 would affect the morphology of macrophages in different polarization states and combined two visualization approaches—fluorescence microscopy and atomic force microscopy (AFM)—to assess these changes.

First, we stained the cells with phalloidin to visualize f-actin (Figure S3). As the staining pattern of phalloidin is uniform and is observed in all cell types, we used it as a mask to define the cell outlines. These images were used to determine the mean circularity and elongation factor of cells.

Circularity was calculated using the following Equation (1):

(1) $$Circularity=\frac{4\pi \times A}{P^{2}},$$

where *A* is the area of the object and *P* is the perimeter of the object.

The elongation factor was calculated using the following Equation (2):

(2) $$Elongation\;factor=\frac{Major\;axis\;length}{Minor\;axis\;length},$$

where the Major axis length is the longest axis of the object that passes through the center of the object and extends from one end to the other, and the Minor axis length is the shortest axis of the object; it also passes through center of the object and is perpendicular to the major axis [35].

**Figure 5:**
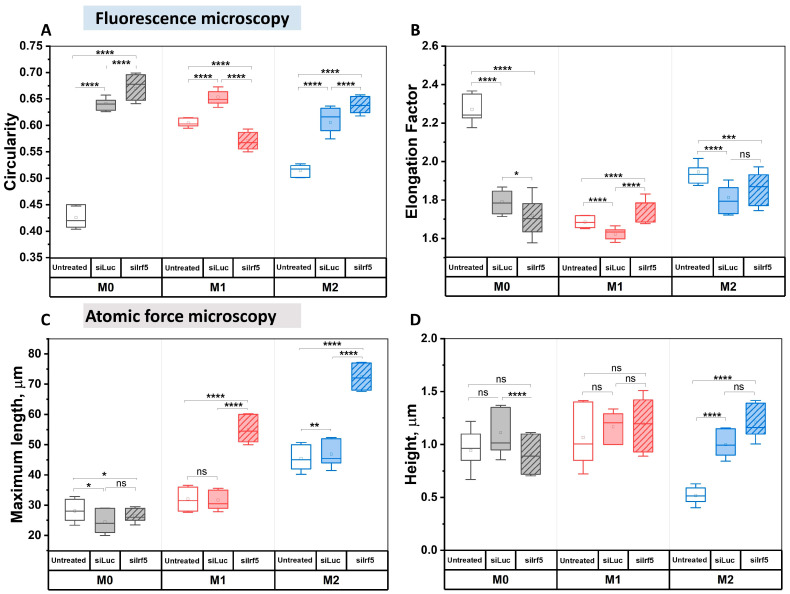
Morphological characterization of macrophages following Irf5 knockdown. Analysis of macrophage circularity (**A**), elongation factor (**B**), maximum length (**C**) and height (**D**) in M0, M1 and M2 states under different conditions (Untreated, siLuc-treated and siIrf5-treated). Mann–Whitney U test, ns—not significant, * *p* < 0.05, ** *p* < 0.01, *** *p* < 0.001, **** *p* < 0.0001.

The analysis of fluorescent images revealed that circularity of all cell types increased in response to treatment with siLuc, especially in M0 macrophages. However, after treatment with siIrf5, the circularity of M1 macrophages decreased, while the circularity of M0 and M2 macrophages increased further (Figure 5A). Moreover, treatment with either siLuc or siIrf5 caused a decrease in the elongation factor in all groups of cells with maximal effect in untreated M0. Treatment with siIrf5 caused a further decrease in the elongation factor of M0 macrophages; however, in M1 and M2 macrophages, the elongation factor increased moderately (Figure 5B).

**Figure 6:**
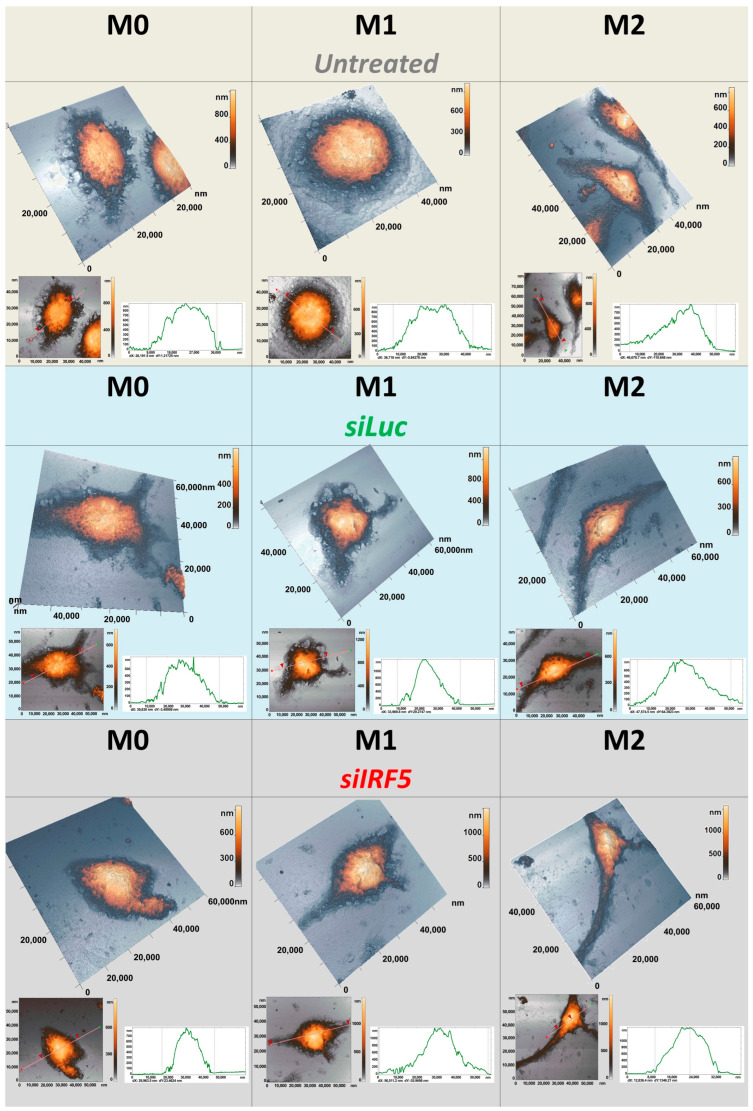
AFM images of M0, M1 and M2 macrophages: non-transfected (beige background), siLuc-treated (light blue background) and siIrf5-treated (grey background). Images from each group are presented as 3D and 2D height maps and cell surface roughness profiles are shown for each cell type.

Next, we acquired AFM images of individual cells, which were used to estimate cell height and the maximum cell length. The M2 macrophages had the greatest maximum cell length (45.0 ± 7.0 μm), while the maximum cell length of M1 macrophages was more similar to the one of M0 macrophages (32.0 ± 6.0 μm and 28.0 ± 7.0 μm, respectively). Also, the height of M2 macrophages (0.51 ± 0.20 μm) was significantly lower as compared to M0 and M1 macrophages from the same group (0.94 ± 0.46 μm and 1.06 ± 0.51 μm, respectively). Treatment with siLuc did not have a strong effect on the maximum cell length in all groups, with only a slight decrease in M0 macrophages and a slight increase in M2 macrophages. The height of M0 cells that were treated with siLuc, as measured in the profile, was 1.10 ± 0.30 μm, with the maximum length being within the range of 24.0 ± 6.0 μm. The height of M1 cells that were treated with siLuc, as measured in the profile, was approximately 1.15 ± 0.25 μm, and its maximum length was from 31.0 ± 5.0 μm. The maximum cell length remained the highest in M2 macrophages—46.0 ± 8.0 μm. We also observed an increase in the height of M2 macrophages (1.01 ± 0.15 μm); however, it still appeared slightly lower as compared to M0 and M1 macrophages from the same group (Figure 6).

In contrast, treatment with siIrf5 caused a strong increase in the maximum cell length of M1 and M2 macrophages. The height of siIrf5-treated M1 macrophages, as measured in the profile, was 1.20 ± 0.5 μm, with the maximum cell length of 55.0 ± 7.0 μm (Figure 6). The maximum length of M0 and M2 macrophages were 26.0 ± 4.0 μm and 72.0 ± 8.0 μm, respectively. Within this group, the height of M0 macrophages appeared to be the lowest (0.91 ± 0.28 μm), while the heights of M1 and M2 macrophages were similar (1.20 ± 0.50 μm and 1.21 ± 0.20 μm, respectively). Transfection with siIrf5 did not cause any significant changes in the height of M1 and M2 macrophages as compared to siLuc, while the height of M0 macrophages decreased slightly (Figure 5C,D). Taken together, these results show that suppression of Irf5 promotes the elongation of the shape of M1 macrophages, and makes them morphologically resemble M2 phenotype.

### 3.4. Irf5 Knockdown Was Associated with Increased Plasma Membrane Roughness in M2-Polarized BMDM

**Figure 7:**
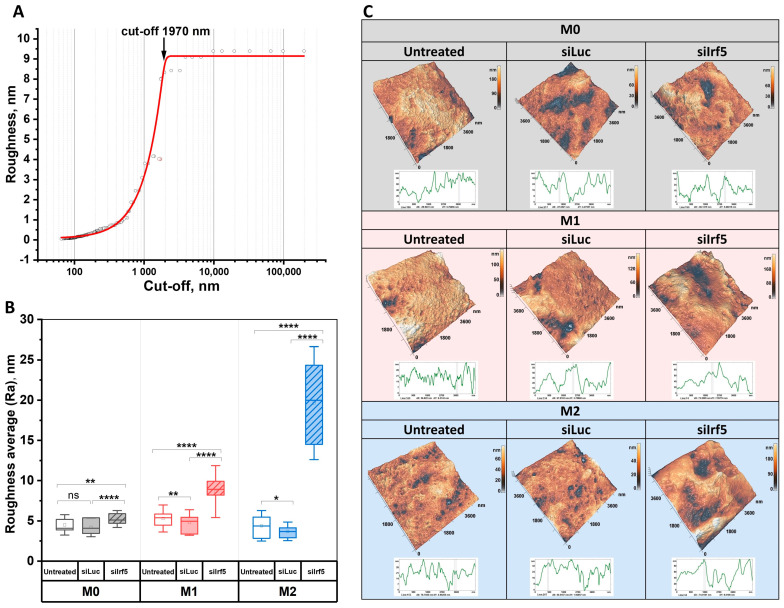
Analysis of macrophage membrane nanostructure. Derivative roughness function (**A**). Roughness average of M0, M1 and M2 macrophages (**B**). Images of macrophage nanostructure: non-transfected, siLuc-transfected and siIrf5-transfected M0 (grey background), M1 (red background) and M2 (blue background) macrophages (**C**). Mann–Whitney U test, ns—not significant, * *p* < 0.05, ** *p* < 0.01, **** *p* < 0.0001.

The nanostructure of macrophage plasma membrane plays a critical role in their biological functions, influencing processes like cell signaling, adhesion, and phagocytosis [36,37]. To assess the nanostructural changes, we acquired AFM images of M0, M1 and M2 macrophages from each experimental group (Figure 7).

The cut-off parameter used in spatial roughness value calculations specifies the maximum lateral dimension for determining local surface roughness (Figure 7A). Analyzing the variation in roughness values with different cut-off parameter values enabled identification of the inflection point in the roughness derivative function (Figure S4). Assessing the roughness of AFM images at the selected cut-off parameter value (1970 nm) enhanced sensitivity to external influences on surface characteristics. Moreover, this cut-off value ensured consistent evaluation across all cell images obtained in this study, including those captured at lower resolution.

Treatment with siLuc caused a slight decrease in the average roughness (Ra) of M1 and M2 macrophages, while the Ra of M0 macrophages did not change significantly. However, treatment with siIrf5 caused an increase in the Ra of all cell types, especially in M2 macrophages (Figure 7B). These results demonstrate the essential role of IRF5 in maintaining the structural organization of the plasma membrane. Knockdown of Irf5 is associated with dramatic changes in plasma membrane landscape, increasing its roughness in both M1 and M2 macrophages, with a more profound effect in M2 (Figure 7C).

### 3.5. The Stiffness of M1 Macrophages Increases in Response to Irf5 Knockdown

**Figure 8:**
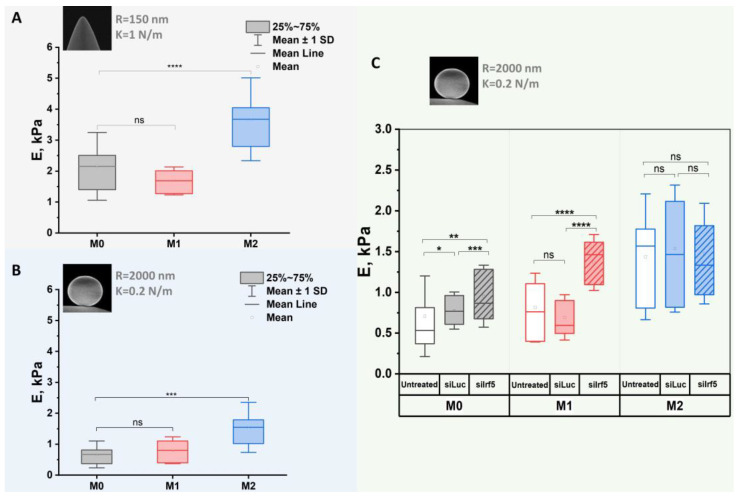
Young’s modulus values of untreated M0, M1 and M2 macrophages measured with conical (**A**) and spherical (**B**) AFM probes (measurements: n = 50 per group). Changes in Young’s modulus values of M0, M1 and M2 macrophages after treatment with siLuc and siIrf5: measurements (n = 50 per group) performed using spherical AFM indenters (**C**). Mann–Whitney U test, ns—not significant, * *p* < 0.05, ** *p* < 0.01, *** *p* < 0.001, **** *p* < 0.0001.

Previous studies have demonstrated that cell stiffness directly affects the ability of macrophages to migrate, phagocytize pathogens, and interact with other cells and the extracellular matrix [38,39]. In our study, we assessed the mechanical stiffness of macrophages after treatment with siLuc and siIrf5, with the Young’s modulus being the measure of elasticity (Figure 8).

Initially, we used two different probes-conical and spherical AFM indenters-to measure the elastic modulus of untreated M0, M1 and M2 macrophages. The measurements performed using a conical probe did not reveal any significant differences between the average Young’s modulus of M0 and M1 macrophages. In contrast, the Young’s modulus of M2 macrophages was significantly higher (Figure 8A). When the stiffness measurements were performed with a spherical probe, the trend in Young’s modulus values among M0, M1 and M2 groups was consistent with the results obtained using the conical probe. However, the overall Young’s modulus values were lower (Figure 8B). The lower Young’s modulus values were likely observed due to the fact that the spherical probe creates a larger contact area with the cell surface. This decreases the magnitude of local stress and thus avoids nonlinearity in the behavior of the strain stress, which leads to an overestimation of the elastic modulus value, which is critical for accurate cell stiffness measurement [40,41]. Nevertheless, despite the difference in the values obtained, the relative difference in the Young’s modulus between the M0, M1 and M2 groups remained similar for both probe types.

Next, we used the spherical probe to assess the changes that occurred in M0, M1 and M2 macrophages after the knockdown of Irf5. Following siLuc treatment, M1 and M2 macrophages did not show any measurable changes in the Young’s modulus, whereas M0 macrophages showed a slight increase. However, the Young’s modulus of M0 and M1 macrophages increased in response to treatment with siIrf5, while the Young’s modulus of M2 macrophages did not undergo any significant changes (Figure 8C). These findings support the observations that were described above and show that decreased levels of IRF5 in M1 macrophages increase their stiffness and shift this quantitative trait toward M2 phenotype.

## 4. Discussion and Conclusions

In this study, we examined how IRF5 influences some of the key phenotypic traits of M1 and M2 macrophages, including protein marker expression, cellular morphology and metabolic signatures. Our findings demonstrate that a reduction in the levels of IRF5 in M1 macrophages are associated with a shift toward M2-like characteristics, as evidenced by changes in marker expression, mitochondrial dynamics and some of the mechanical features. Interestingly, according to our results, IRF5 can act both as an inducer and a repressor of the key marker proteins, activating iNOS in M1 and suppressing it in M2; at the same time, it activates CD206, in M2 state and suppresses it in M1. These findings suggest that IRF5 is an important regulator of macrophage plasticity, influencing both pro-inflammatory and anti-inflammatory responses.

The antisense sequence to insect luciferase, siLuc, is used as a standard negative control insiRNA silencing experiments. However, despite the fact that siLuc lacks complementary sequences in mammalian transcriptome, lipofectamine transfection with siLuc oligonucleotide was associated with increased expression of iNOS, which is responsible for the synthesis of nitric oxide from L-arginine. Nitric oxide is a reactive nitrogen species and is an essential effector of pro-inflammatory M1 macrophages, associated with antimicrobial and antiviral activities [42]. It was shown earlier that the lipid nanoparticles, which are used for the encapsulation of the siRNA particles, may elicit an inflammatory response [43]. The results of our earlier study, where we performed siRNA-mediated knockdown of the *Has2* gene in vivo, are in line with this evidence [44]. The effect that we observed in response to siLuc has likely occurred in response to the encapsulating agent. Even though iNOS was upregulated after the administration of siLuc, siRNA-mediated knockdown of the *Irf5* gene significantly reduced the levels of iNOS in M1 macrophages. Similar results were also observed in some of the earlier studies, which described a reduction in the expression of iNOS in BMDM of *Irf5^−/−^* mice after stimulation with lipid A [45]. Meanwhile, CD206 is a C-type lectin receptor that binds to mannose residues on the surface of pathogens to facilitate their recognition and phagocytosis, which is commonly observed in M2 macrophages. In addition to pathogen clearance, CD206 is also involved in the endocytosis of glycoproteins and the modulation of immune responses [46]. M1 macrophages that were transfected with siIrf5 had an increased expression of CD206. Moreover, treatment with siIrf5 induced changes in the morphology of M1 macrophages, where M1 macrophages acquired an elongated and spindle-like shape, which is typical for the M2 phenotype [47]. Our observations align with the findings from the previous studies, which show that Irf5 knockdown alters the expression profile of M1 macrophages, making them more similar to M2 macrophages [15,20]. At the same time, treatment with siIrf5 caused an increase in iNOS and a decrease in CD206 in M2 macrophages. These findings are in line with the results of our earlier study, where we performed a characterization of BMDM after the knockdown of genes that are involved in the regulation of macrophage polarization [20]. Here we show for the first time that IRF5 can activate or inhibit genes specific to M1 or M2 depending on the context of the cell, upregulating iNOS in M1 and downregulating it in M2, or in the reverse manner, stimulating CD206 in M2 and suppressing it in M1. Similar phenomena was reported previously for the M1 state, where IRF5 activated pro-inflammatory cytokines IL-10 and IL-23 while suppressing the anti-inflammatory cytokine IL-10 [5,48]. Here we report the reverse phenomenon in M2 macrophages for the pro-inflammatory marker iNOS and the anti-inflammatory marker CD206.

Another indicator of the shift toward an M2-like phenotype was the increase in the mitochondrial content after Irf5 knockdown. In M1 macrophages, the TCA cycle is disrupted, which leads to an increase in the levels of citrate and succinate [49,50]. Citrate is converted into itaconic acid, which has bactericidal effects, while succinate is directly associated with the expression of pro-inflammatory cytokines [49,51]. These changes contribute to the overall pro-inflammatory phenotype of M1 cells. Importantly, macrophages also require glycolysis for efficient transition into the M2 state [52]. However, IL10, which is directly associated with M2 polarization, acts upon the mTOR pathway by inhibiting mTORC1 through STAT3, which promotes oxidative phosphorylation. Moreover, IL10 is associated with elimination of dysfunctional mitochondria in M2 cells [53]. Therefore, M2 macrophages have a higher mitochondrial content. Our results showed that the knockdown of Irf5 affected all phenotypes. The role of IRF5 in mitochondrial function within macrophages has also been previously documented. IRF5 was shown to promote the expression of glycolysis-related genes through Akt2 signaling [54]. Also, IRF5 was shown to be interacting with GHITM, a protein which is strongly involved in the maintenance of mitochondrial cristae architecture and mitochondrial function. Lack of IRF5 led to impaired interactions with GHITM and subsequent disruption of mitochondria [55]. Interestingly, one of the studies suggested that the association between IRF5 and the metabolic function of macrophages may depend on the subset of macrophages; knockout of Irf5 was shown to impact the metabolism of BMDM, while the airway macrophages were not affected by the knockout [56]. These results highlight the importance of IRF5 in multiple aspects of macrophage homeostasis and function.

Furthermore, our findings suggest that IRF5 plays an essential regulatory role in the biomechanical state of macrophages. Treatment with siIrf5 induced notable alterations in cell morphology, membrane nanostructure and stiffness, particularly in M1 and M2 subtypes. These alterations include increased surface roughness and cell elongation, both of which are associated with the M2 phenotype.

It has been demonstrated that alterations in membrane roughness and stiffness can exert a significant influence on the adhesion, motility, distribution of receptors and phagocytic efficiency of macrophages [38,57,58,59,60]. The enhanced surface roughness observed subsequent to IRF5 knockdown may be indicative of cytoskeletal reorganization and altered receptor dynamics, which could potentially influence CD206-mediated phagocytosis, vesicle secretion, lipid mediator production and antigen processing. Despite the absence of direct functional assays in this study, the findings support the hypothesis that IRF5 modulates macrophage mechanical properties in a manner that could influence its functional properties. As demonstrated in the study by Park, J.S. et al. [61], the disruption of glycolysis results in cytoskeletal remodeling, which may be a contributing factor to the observed changes in membrane roughness and cell stiffness following Irf5 knockdown.

We observed an increase in Young’s modulus in M1 macrophages after being treated with siIrf5, bringing them closer to the stiffer M2 macrophages. Previous studies showed that the macrophage membrane stiffness is influenced by a range of factors, including the substrate stiffness [38,39]. Importantly, under physiological conditions, M2 macrophages often reside within regions where the tissues have a higher density. Changes in mechanical properties may be reflecting the switch in the metabolic and functional states of macrophages. Some of the previous studies also described an increase in the stiffness of human monocytes that were polarized to M2 state, suggesting that the interaction of M2 macrophages with rigid surfaces promotes the production of f-actin, ultimately leading to a decrease in membrane elasticity [62]. These results are consistent with the findings showing that M2 macrophages have higher levels of f-actin as compared to M0 and M1 macrophages [47]. Moreover, it has been suggested that the mechanical properties of macrophages are related to their immune function [38,63,64]. The increased surface roughness of macrophages observed after siIrf5 transfection indicates structural rearrangements at the membrane level, which is consistent with the data on the significant effect of cytoskeletal changes on the morphology and functional state of cells [65,66].

In summary, our data illustrate the importance of IRF5 in the modulation of phenotypic plasticity of macrophages. By performing siRNA-mediated knockdown of *Irf5*, we demonstrated complex changes in macrophage phenotype, including the changes in marker expression, mitochondrial content and biomechanical properties such as cell stiffness and surface roughness.

These findings offer novel insights into the mechanobiological role of IRF5 in regulating macrophage phenotype and suggest that the mechanical properties of the cell may serve as functionally relevant indicators of the state of cellular polarization.

Importantly, these findings highlight the complexity of macrophage biology and suggest that IRF5 plays a context-dependent role in their metabolic and functional programming. We show that polarization of macrophages toward specific states through elimination of IRF5 presents a promising therapeutic strategy for diseases that are driven by dysregulated inflammation.

Recently transplantation of autologous macrophages was shown to be safe and efficacious in the phase I and phase II clinical trials [67]. The suggested development of this approach will be utilization of “engineered” M2 macrophages polarized by cytokines and enhanced by genetic modifications [68]. We believe that polarization or “engineering” of macrophages toward pro regenerative M2 phenotype with siRNA-mediated knockdown of Irf5 will be safer, more efficacious and long-lasting.

## Data Availability

The original contributions presented in this study are included in the article/Supplementary Materials. Further inquiries can be directed to the corresponding authors.

## Supplementary Materials

The following supporting information can be downloaded at: https://www.mdpi.com/article/10.3390/cells15030238/s1, Figure S1: Representative fluorescent images of non-transfected (M0: n = 10, M1: n = 10, M2: n = 10, (A)), siLuc-transfected (M0: n = 10, M1: n = 10, M2: n = 10, (B)) and siIrf5-transfected macrophages (M0: n = 10, M1: n = 10, M2: n = 10, (C)) stained with antibodies against iNOS (left panel, green) and CD206 (right panel, red). Magnification: ×20, scalebar = 50 µm. Figure S2: Representative images of live cells stained with fluorescent MitoFlamma Green dye. Brightfield (grey) and fluorescent images (green) of non-transfected (M0: n = 10, M1: n = 10, M2: n = 10, (A)), siLuc-transfected (M0: n = 10, M1: n = 10, M2: n = 10), (B) and siIrf5-transfected (M0: n = 10, M1: n = 10, M2: n = 10, (C)) macrophages. Magnification: ×20, scalebar = 50 µm. Figure S3: Representative images of non-transfected (M0: n = 10, M1: n = 10, M2: n = 10, (A)), siLuc-transfected (M0: n = 10, M1: n = 10, M2: n = 10, (B)) and siIrf5-transfected macrophages (M0: n = 10, M1: n = 10, M2: n = 10, (C)) stained with fluorescent phalloidin stain (green). Magnification: ×40, scalebar = 50 µm. Figure S4: Dependence of derivative of roughness function on cut-off value. Maximum of derivative at 1970 nm corresponds to the point where the surface roughness value is most sensitive to structural changes in cells. Figure S5: Dose-response of Irf5 knockdown in RAW264.7 cells. The relative expression of Irf5 mRNA was measured in cells treated with serial dilutions (from 10 nM to 5 pM) of two siRNAs targeting IRF5 (siIRF5-3 and siIRF5-5) to determine their IC_50_ values for knockdown efficiency. The calculated IC_50_ was (A) 25 pM for siIRF5-3 and (B) 60 pM for siIRF5-5 (n = 3; mean ± SD). For next work, we used Irf5-3 siRNA.
