# Supplementary material for: Irf5 Knockdown in Bone Marrow-Derived Macrophages Favors M1-to-M2 Transition
Source: Cells. 2026 Jan 26;15(3):238. doi: 10.3390/cells15030238 (PMC12897094; doi:10.3390/cells15030238)
Supplement: Supplementary file 1 [file cells-15-00238-s001.zip › cells-3581457-supplementary.pdf]

Supplementary information for

Irf5 knockdown in bone marrow-derived macrophages favors M1-to-M2 transition

Elizaveta Petrova, Ekaterina Sherstyukova, Snezhanna Kandrashina, Vladimir Inozemtsev, Alexandra Tsitrina, Viktoriya Fedorova, Mikhail Shvedov, Artem Kuzovlev, Maxim Dokukin, Yuri Kotelevtsev, Arsen Mikaelyan and Viktoria Sergunova

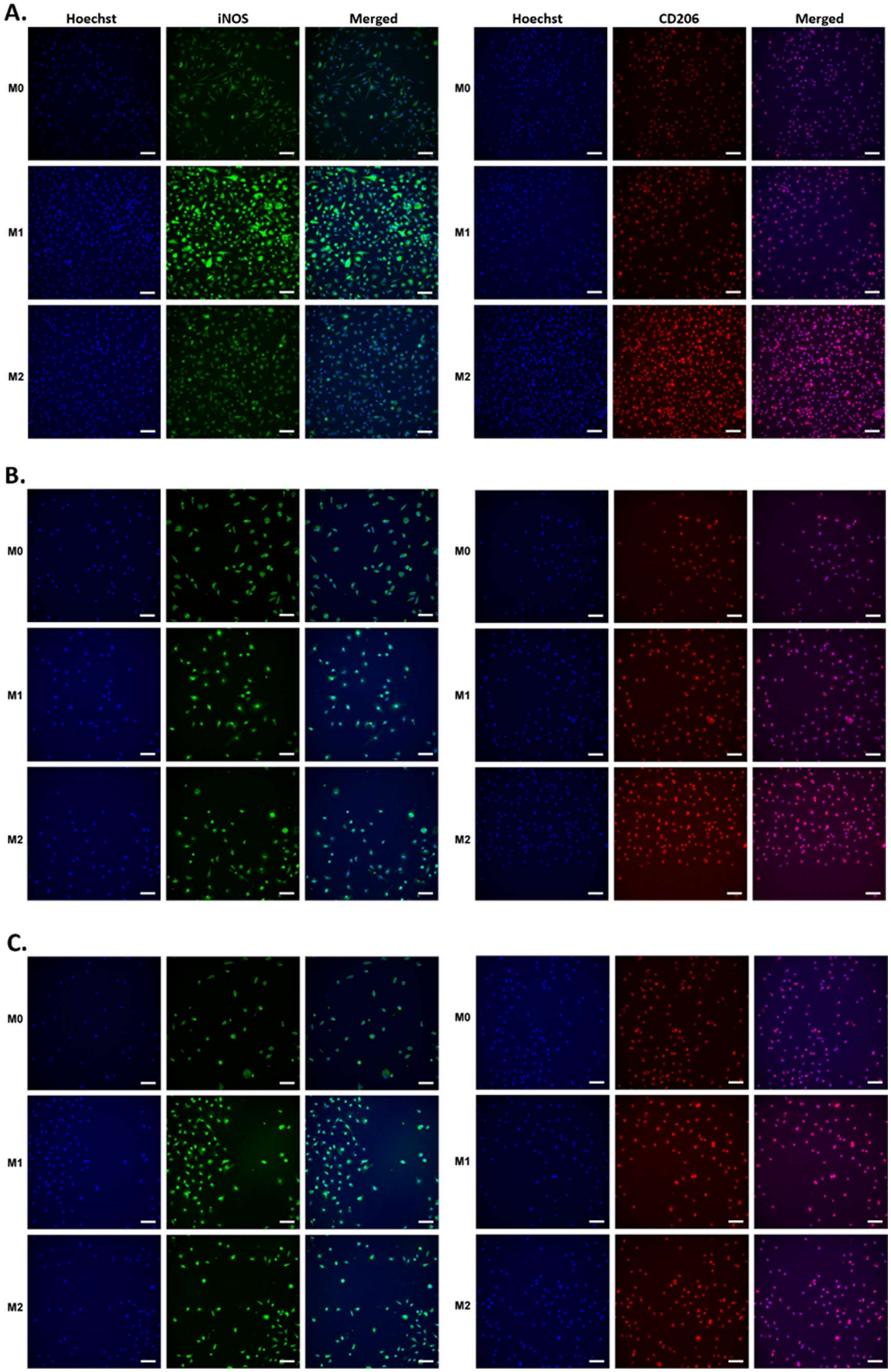

Figure S1. Representative fluorescent images of non-transfected (M0: n = 10, M1: n = 10, M2: n = 10, **A**), siLuc-transfected (M0: n = 10, M1: n = 10, M2: n = 10, **B**) and siIrf5-transfected macrophages (M0: n = 10, M1: n = 10, M2: n = 10, **C**) stained with antibodies against iNOS (left panel, green) and CD206 (right panel, red). Magnification: x20, scalebar = 50 µm.

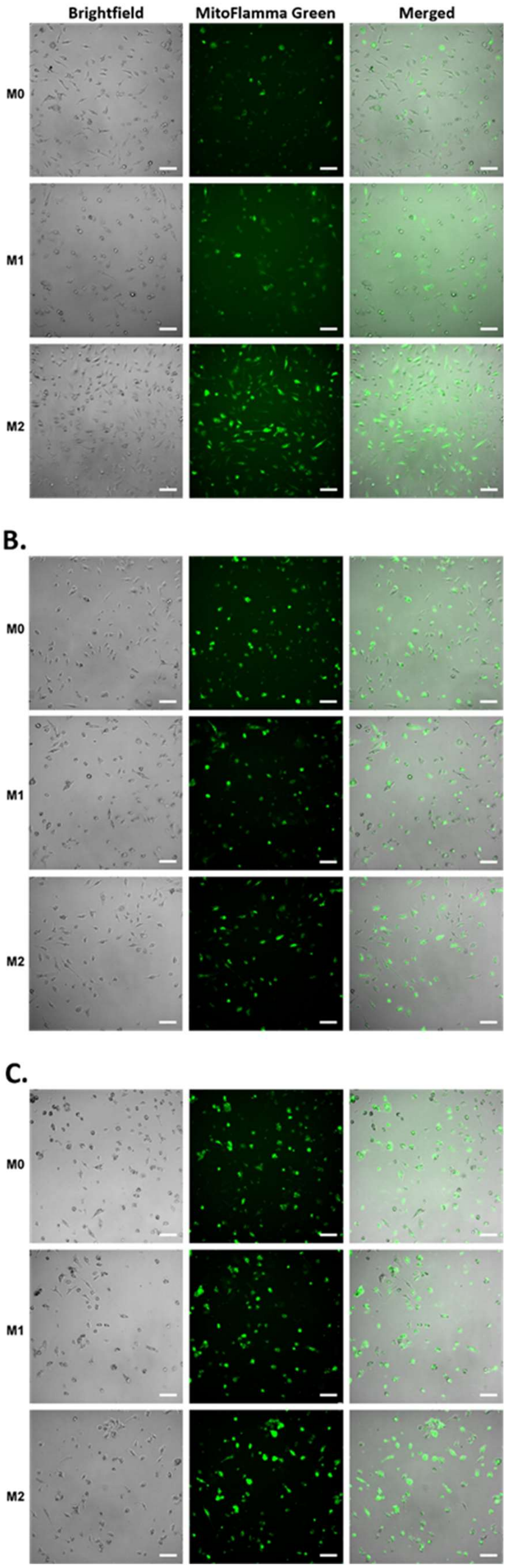

Figure S2. Representative images of live cells stained with the fluorescent MitoFlamma Green dye. Brightfield (grey) and fluorescent images (green) of non-transfected (M0: n = 10, M1: n = 10, M2: n = 10, **A**), siLuc-transfected (M0: n = 10, M1: n = 10, M2: n = 10), **B**, and siIrf5-transfected (M0: n = 10, M1: n = 10, M2: n = 10, **C**) macrophages. Magnification: x20, scalebar = 50 µm.

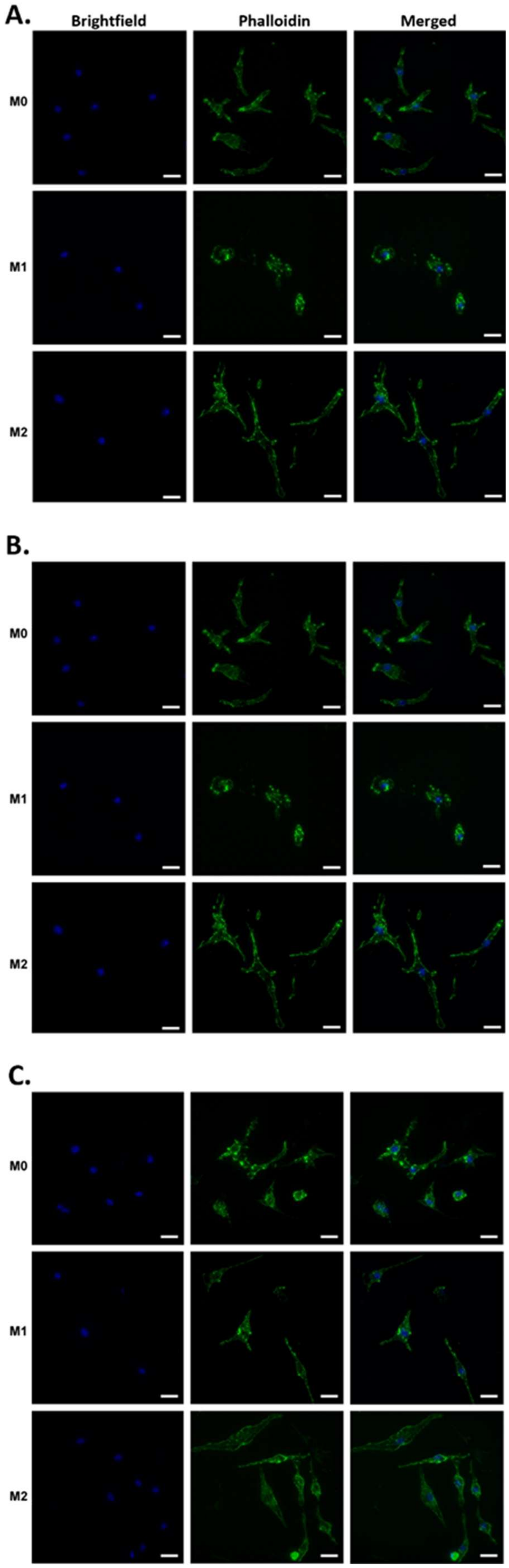

Figure S3. Representative images of non-transfected (M0: n = 10, M1: n = 10, M2: n = 10, **A**), siLuc-transfected (M0: n = 10, M1: n = 10, M2: n = 10, **B**) and siIrf5-transfected macrophages (M0: n = 10, M1: n = 10, M2: n = 10, **C**) stained with the fluorescent phalloidin stain (green). Magnification: x40, scalebar = 50 µm.

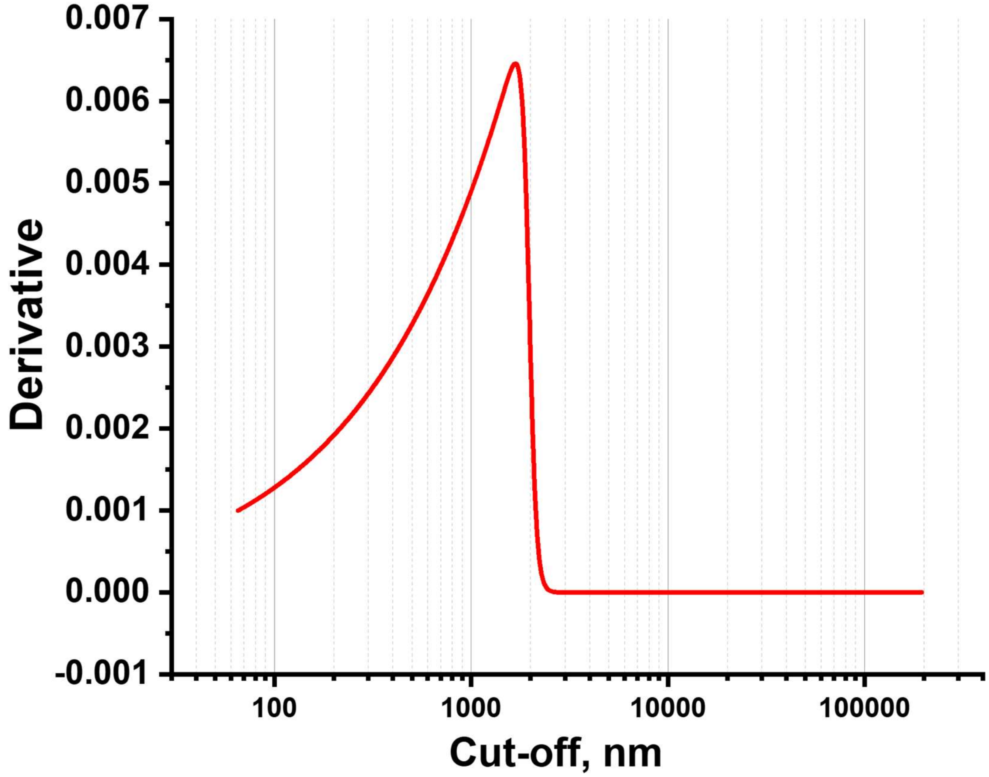

Figure S4. The dependence of the derivative of the roughness function on the cut-off value. The maximum of the derivative at 1970 nm corresponds to the point where the surface roughness value is most sensitive to structural changes in cells.

Irf5-mediated knockdowns in RAW 264.7 cells.

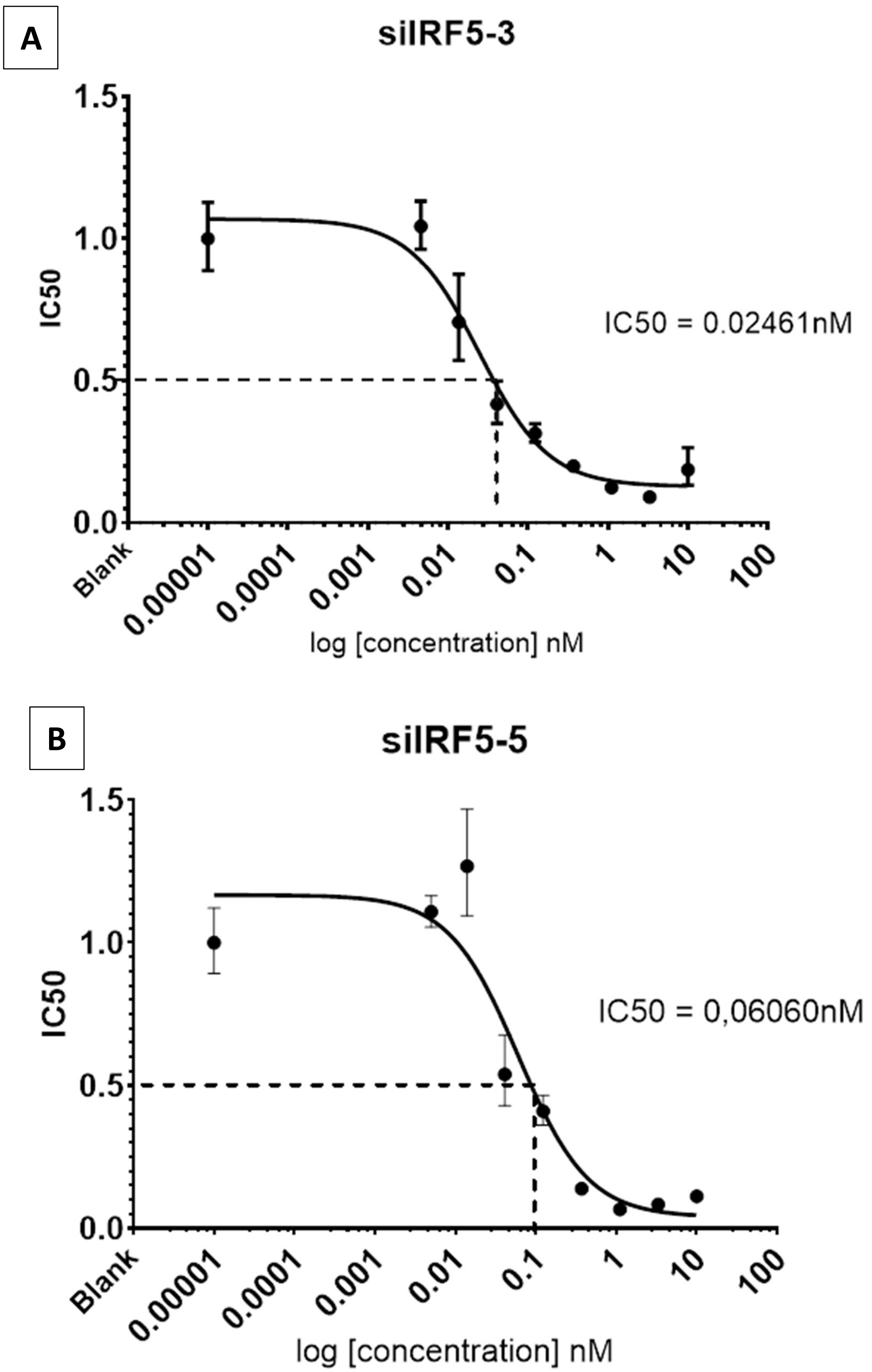

Figure S5. Dose-response of Irf5 knockdown in RAW264.7 cells. The relative expression of *Irf5* mRNA was measured in cells treated with serial dilutions (from 10 nM to 5 pM) of two siRNAs targeting IRF5

(siIRF5-3 and siIRF5-5) to determine their IC_{50} values for knockdown efficiency. The calculated IC_{50} was (A) 25 pM for siIRF5-3 and (B) 60 pM for siIRF5-5 (n=3; mean±SD).

For next work, we used Irf5-3 siRNA.
